# Risk of Breast and Ovarian Cancer After Prophylactic Mastectomy and Salpingo‐Oophorectomy in BRCA1/2 Germline Variant Carriers: A Retrospective Cohort Study From a Single German Center

**DOI:** 10.1002/cam4.71691

**Published:** 2026-03-09

**Authors:** Sara Mendes, Maryam Yahiaoui‐Doktor, Eva Maria Fallenberg, Marion Kiechle, Sabine Grill

**Affiliations:** ^1^ Department of Gynecology and Obstetrics, University Hospital Klinikum Rechts der Isar Technical University Munich (TUM) Munich Germany; ^2^ Institute for Medical Informatics, Statistics and Epidemiology University of Leipzig Leipzig Germany; ^3^ Department of Diagnostic and Interventional Radiology, University Hospital Klinikum Rechts der Isar Technical University of Munich (TUM) Munich Germany

**Keywords:** cancer prevalence, gPV BRCA1/2 germline variants, hereditary breast and ovarian cancer, prophylactic mastectomy, prophylactic salpingo‐oophorectomy

## Abstract

**Background:**

As the data on *BRCA1/2*‐associated breast and ovarian cancer prevalence after prophylactic surgery has not been exhaustively investigated yet, we aimed to evaluate the cancer prevalence in a single center cohort of *BRCA1* and *BRCA2* carriers after conducting prophylactic mastectomy, as well as prophylactic bilateral salpingo‐oophorectomy (PBSO) respectively.

**Methods:**

We included 875 women that were tested positive for a germline variant in the *BRCA1/BRCA2* gene (gPV) between 2002 and 2022 at the Center of Hereditary Breast and Ovarian Cancer of the Technical University Munich Germany. Mean follow up was 7.2 years (range 0–44 years; 95% CI: 6.70 to 7.70). We differentiated breast and/or ovarian cancer diseased (*n* = 643) and non‐diseased *BRCA1/2* carriers (*n* = 232).

**Results:**

Our analysis confirmed the effectiveness of prophylactic surgeries in genetically predisposed women with a gPV in the *BRCA1*/2 gene. We observed no breast cancer after prophylactic bilateral mastectomy, 2 contralateral breast cancer diseases after contralateral prophylactic mastectomy and 1 extraovarian serous adenocarcinoma after PBSO. Within the entire study collective, a total of 293 have undergone PBSO, with 6 women having an incidental finding of ovarian cancer and STIC respectively (=2.0%; 1.7% *gBRCA1* and 0.3% *gBRCA2*). Our data suggests that, particularly regarding ipsilateral secondary cancer (ISC), higher oncological safety can be achieved through mastectomy rather than breast‐conserving surgery (BCS). In the group of patients who had a second breast cancer and were treated with BCS during their first cancer, 18.3% showed an ISC. Within the patients who were first treated with a mastectomy, only 4.3% showed an ISC.

**Conclusions:**

Prophylactic surgeries demonstrate high oncological effectiveness in gPV *BRCA1/2* carriers. In particular, mastectomy may provide greater protection against ISC compared with BCS. Further studies will have to be conducted to compare ipsilateral cancer prevalence after breast‐sparing surgery and mastectomy.

## 1 Introduction

Breast cancer is the most common cancer among women with an evaluated lifetime risk of 13.2% [1]. Familial susceptibility to breast cancer thereby accounts for approximately 30%–40% of all breast cancer cases. An estimated 10% of all breast cancers are developed monogenic, meaning that a single disease‐causing (pathogenic) variant is responsible for the increased risk. The majority of the monogenic inherited breast cancer diseases is attributed to the tumor suppressor genes *BRCA1* and *BRCA2*, which play a crucial role in DNA double‐strand repair (homologous recombination). Pathogenic germline variants (gPV) in these genes confer a high risk to breast and ovarian cancer, respectively [2]. The cumulative lifetime risk of developing breast cancer and ovarian cancer is 72% and 44% for *BRCA1*, as well as 69% and 17% for *BRCA2*, respectively [3]. Given these high cancer risks, carriers of a pathogenic (g) *BRCA1/BRCA2* variant are opting to take part in an intensified breast cancer screening program or optionally undergo prophylactic surgeries, including prophylactic mastectomy (PBM) and bilateral salpingo‐oophorectomy (PBSO). The German Consortium for Hereditary Breast and Ovarian Cancer recommends that carriers of a gPV in *BRCA1/2* participate in an intensified breast cancer screening program from the age of 25, or 5 years before the earliest breast cancer diagnosis in the family [2]. The screening program consists of an annual MRI and six‐monthly sonography [2]. From the age of 40, mammography can be performed additionally every 1–2 years according to physicians' choice [2]. Out of these diagnostic tools, MRI is the most sensitive screening method for early breast cancer detection [4, 5, 6].

By contrast, early detection of ovarian cancer is still insufficient [7, 8, 9]. Neither screening with serum CA125 concentration regardless of whether it is examined serially or one‐time, nor transvaginal ultrasound screening resulted in mortality reduction [7, 8, 9].

Due to the high lifetime risks of developing breast and ovarian cancer, a great share of these carriers opt for a primary preventive approach of prophylactic surgery, primarily PBSO, as effective screening options for early detection of ovarian cancer are still missing. PBSO can reduce the risk of developing ovarian cancer by 80%–90% [10, 11, 12, 13, 14]. Under consideration of the age‐dependent risk for ovarian cancer and the postoperative side effect of premature menopause, the procedure is currently recommended from the age of 40 years, or 5 years before the youngest age of onset of ovarian cancer [12, 15]. In exceptional cases carriers of a gPV in *BRCA1* can undergo the procedure from the age of 35 [15]. As far as prophylactic mastectomy is concerned, several studies have shown that PBM significantly reduces the incidence of breast cancer by 90%–100% [14, 16, 17, 18, 19, 20, 21]. Once PBM is conducted, the determined residual lifetime risk for breast cancer is given 4%–6%. In light of this dramatic risk reduction, the intensified screening program will be terminated.

As the data on *BRCA1/2*‐associated breast and ovarian cancer prevalence after prophylactic surgery has not been exhaustively investigated yet, we aimed to evaluate the cancer prevalence in a single center cohort of *BRCA1* and *BRCA2* carriers after conducting prophylactic bilateral and contralateral mastectomy, as well as prophylactic bilateral salpingo‐oophorectomy, respectively.

## 2 Results

A total of 875 female patients were included in the analysis. All had been tested positive for a gPV in the genes *BRCA1* or *BRCA2* at the FBREK center TU Munich, Germany.

**FIGURE 1:**
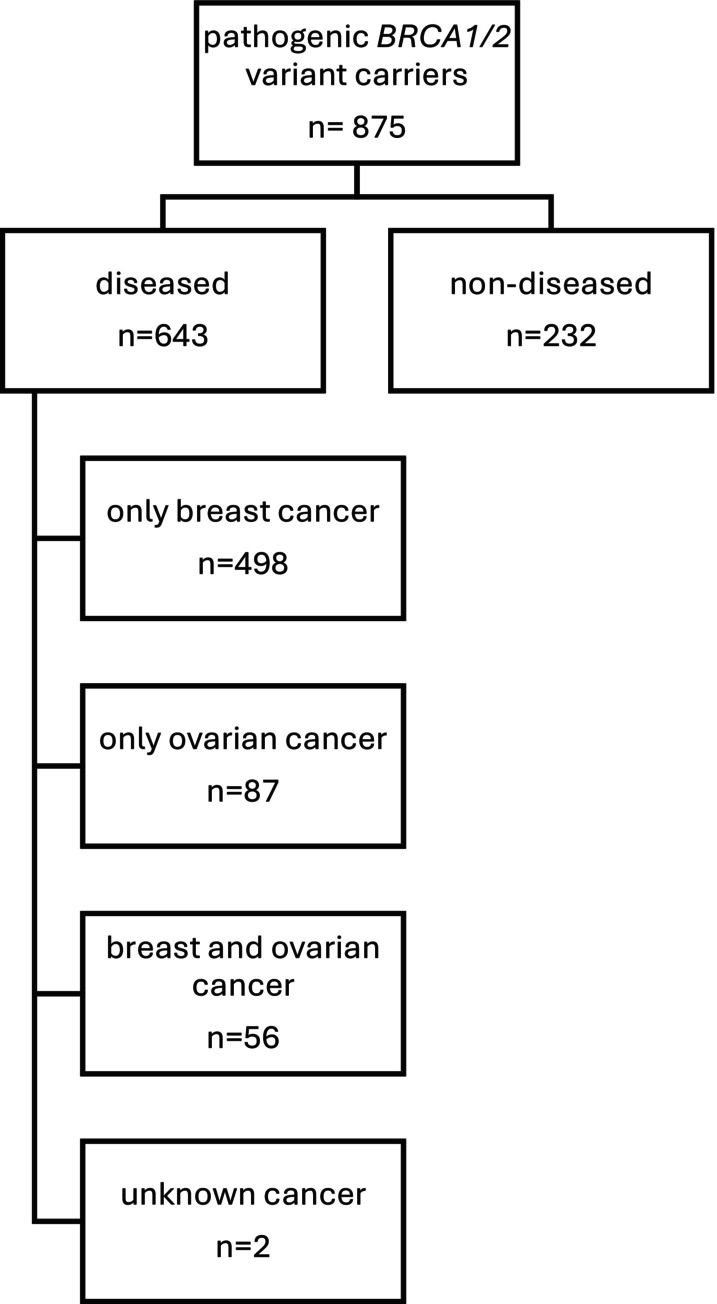
Flowchart of study cohort classification. The diagram shows the distribution of 875 individuals with pathogenic BRCA1/2 variants, categorized into diseased (*n* = 643) and non‐diseased (*n* = 232). Diseased individuals were further classified based on cancer type: only breast cancer (*n* = 498), only ovarian cancer (*n* = 87), both breast and ovarian cancer (*n* = 56), and unknown cancer (*n* = 2).

For analysis we differentiated diseased (breast and/or ovarian cancer; *n* = 643) and non‐diseased *BRCA1/2* carriers (*n* = 232) (Figure 1).

### 2.1 Diseased Cohort

Within the cohort of diseased participants (*n* = 643) 61.6% were tested positive for a gPV in *BRCA1* and 37.9% for *BRCA2* respectively. Only 0.2% (*n* = 1) of study participants carried a gPV in both genes. The prevalence of breast cancer was 86.2% and 22.2% for ovarian cancer. 10.1% of patients were affected by both cancers. *BRCA1* carriers were diagnosed at younger age compared to participants with a gPV in the *BRCA2* gene (41.6 vs. 46.1 years for breast cancer. *p* < 0.001; 53.5 vs. 60.4 years for ovarian cancer, *p* < 0.001). The presence of multiple tumor diseases was almost doubled in g*BRCA1* carriers with a rate of 10.6% compared to 5.7% in *gBRCA2* carriers. All analyses in the diseased cohort refer to a mean follow up of 8.2 years (range 0–44 years).

#### 2.1.1 Breast Cancer

Five hundred and fifty‐four study participants are breast cancer diseased with a mean age of 43.4 years at time of diagnosis. 89.3% had unilateral and 4.7% bilateral breast cancer at time of first diagnosis (6% unknown localization). Complete data on hormone receptor and Her2 status was available for 412 patients. A total of 55.3% (*n* = 228) demonstrated triple negative receptor status (TNBC). 85.1% of the triple negative tumors were linked to a gPV in *BRCA1*. As TNBC is a testing criterion for gPV *BRCA1/2* in Germany and often attributed to a gPV in *BRCA1*, it can be expected that this number is generally overestimated. 36.4% (*n* = 150) of the breast cancer cases showed hormone receptor positive and Her2 negative tumor biology, mainly associated with g*BRCA2* carrier status (30.0% *BRCA1* and 70.0% *BRCA2*). The rate of Her2 positive receptor status was limited to 8.3%.

With regard to therapeutical management, data was available for 461 patients. 57.3% (*n* = 264) of the patients opted for a breast‐conserving surgery, and 42.7% (*n* = 197) for a mastectomy. 44.2% (*n* = 87) of the mastectomized patients chose to undergo an additional contralateral prophylactic mastectomy (CPM) in the following 5 years. The mastectomy rate was higher among *BRCA1* carriers compared to patients with a gPV in *BRCA2* (45.4% vs. 38.9%; *p* = 0.171).

Within the collective of the breast cancer diseased carriers, who initially decided on breast‐conserving surgery (*n* = 264), only 14.0% (*n* = 37) of patients chose to undergo CPM in the following 5 years. 23.1% (*n* = 61) subsequently opted for a secondary unilateral mastectomy. On average, this surgery was performed 5.7 years (SD = 5.80) after the initial breast cancer diagnosis at a mean age of 46.57 years (SD = 9.96).

A second breast cancer disease occurred in 30.3% of patients (*n* = 168) after a mean period of 7.8 years (SD = 5.67). Contralateral secondary cancer was most common with a share of 67.3%. 19.7% had an ipsilateral recurrence and 13.0% an ipsilateral secondary cancer. 65.5% were g*BRCA1* and 33.9% were g*BRCA2* carriers. In relation to the total diseased cohort, 17.9% of all *gBRCA1* and 15.2% of all *gBRCA2* carriers developed a contralateral secondary cancer. For 129 patients we had complete data on the type of surgery for secondary cancer treatment. 44.2% opted for breast‐conserving surgery, 49.6% for a mastectomy and 4.7% for a lumpectomy after a previous mastectomy (ipsilateral disease). 2 cases of contralateral breast cancer (1.4%) were reported after CPM. These cases appeared 6 and 8 years after CPM and were associated to a PV in the *BRCA1* gene.

We analyzed the localization of a secondary breast cancer in relation to the type of surgery during the first breast cancer. In this cohort of patients with secondary breast cancer (*n* = 152), 30.9% had undergone a mastectomy and 69.1% a breast‐conserving surgery (*p* < 0.001). Consequently, we looked at the secondary cancer localization (ipsilateral or contralateral). In the group of patients who had breast‐conserving surgery during their first cancer treatment, 19.2% had an ipsilateral recurrence and 18.3% an ipsilateral secondary cancer with a different tumor biology. 62.5% developed a contralateral secondary cancer. Within the patients who were first treated with a mastectomy, 23.4% showed an ipsilateral recurrence, 4.3% an ipsilateral secondary cancer and 72.3% a contralateral secondary cancer. Patients who had had a breast‐conserving surgery tended to have a higher percentage of secondary ipsilateral cancer than the group who had had a mastectomy, yet statistical significance was not reached (37.5% vs. 27.7%; *p* = 0.255). The lower rate in contralateral secondary cancers after breast‐conserving surgery compared to mastectomy could be attributed to scattered radiation after breast‐conserving therapy, though no statistically significant difference (*p* = 0.239) could be found (Table 1).

**TABLE 1:** Second breast cancer localization after mastectomy and breast‐sparing surgery respectively.

		Second BC: cancer localization			Total
		Ipsilateral recurrence	Ipsilateral secondary cancer	Contralateral secondary cancer	
First BC: type of surgery	Breast‐conserving surgery	20	19	65	104
		19.2%	18.3%	62.5%	100.0%
	Mastectomy	11	2	34	47
		23.4%	4.3%	72.3%	100.0%
Total		31	21	99	151
		20.5%	13.9%	65.6%	100.0%

A total of 31 patients (5.6%) developed a third breast cancer (*n* = 21 *BRCA1* gPV; *n* = 10 *BRCA2* gPV). Of these, 6 patients (1.1%) also suffered a fourth breast cancer (*BRCA1* carrier: *n* = 4; *BRCA2* carrier: *n* = 2). On average a third cancer appeared 11.2 years (SD = 6.68) and a fourth cancer 13.5 years (SD = 7.15) after the first cancer.

#### 2.1.2 Ovarian Cancer

In our analysis 143 patients had ovarian cancer. The mean age at the diagnosis was 55.5 years (SD = 9.5). 71.3% were g*BRCA1‐* and 28.7% g*BRCA2‐*carriers.

We noted 6 incidental findings of cancer when conducting PBSO. Out of these 6 patients, 4 patients were diagnosed with early‐stage ovarian cancer (max. pT1c), one patient with serous tubal intraepithelial carcinoma (STIC), and another one with carcinoma of unknown histopathology. Five patients carried a *gBRCA1* and one patient a *gBRCA2*. The mean age at diagnosis was 55.0 years (range 48–60). Out of these patients, 4 had previously been diagnosed with breast cancer.

#### 2.1.3 Prophylactic Bilateral Salpingo‐Oophorectomy

In the breast cancer diseased group (*n* = 554) 39.7% underwent a PBSO with no pathological result. Of these, 55.5% were carriers of a gPV in *BRCA1* and 44.1% in *gBRCA2* (*p* = 0.047). Mean age at the intervention was 48.9 years (SD = 10.10) (mean age *gBRCA1* 47.7 years (*n* = 112; SD = 9.9)), *gBRCA2* 50.4 years (*n* = 91; SD = 10.2).

### 2.2 Non‐Diseased Mutation Carriers

Among the non‐diseased carriers (*n* = 232) 59.9% carried a gPV in *BRCA1* (*n* = 139) and 40.1% in the *BRCA2* gene (*n* = 93). 13.4% opted for a bilateral prophylactic mastectomy exclusively (mean age 31.6 years; age range 19–44; SD = 6.4), 17.7% for PBSO (mean age 45.6 years; SD = 8.251) and 10.8% for both surgeries (mean age PBM 43.4 years; SD = 5.3; PBSO 43.0 years; SD = 7.0). Mean follow‐up was 4.4 years (range 0–19 years).

We did not observe any cases of breast cancer following PBM and only one patient who had a PBSO at the age of 67 developed ovarian cancer by the age of 71 (patient who had a laparoscopic PBSO at 67 due to an ovarian cyst progressive in size and developed an extraovarian serous adenocarcinoma (G3) at the age of 71 with lymphangiosis carcinomatosa and multiple lymph node metastases).

As shown in Table 2, our results suggest that *gBRCA1* carriers undergo PBSO significantly more frequently than *gBRCA2* carriers (72.7% vs. 27.3%; *p* = 0.012). Yet no difference could be found concerning PBM rate (*p* = 0.164).

**TABLE 2:** Non‐diseased gBRCA1/2 carrier status and prophylactic surgeries.

			PBM	PBSO
*gBRCA1/2* carriers (*n* = 232)	*gBRCA1* (*n* = 139)	Count	38	48
		%	67.9%	72.7%
	*gBRCA2* (*n* = 93)	Count	18	18
		%	32.1%	27.3%
Total		Count	56	66
		%	100.0%	100.0%

### 2.3 Cancer Screening Participation

19.3% (*n* = 124) of all patients in the cancer diseased group (*n* = 643) participated in the intensified cancer screening program at rechts der Isar hospital. 14.6% were not included into the program due to bilateral mastectomy and 66.1% did not participate at all or visited a different hospital for the diagnostics. Out of all patients who initially had a breast conserving therapy (*n* = 264), 26.1% participated in the intensified program and 12.1% were excluded due to secondary bilateral mastectomy, when looking at the patients who initially underwent a mastectomy (*n* = 197), 9.6% participated and 30.5% were excluded due to bilateral mastectomy.

In the non‐diseased group (*n* = 232), the participation in the intensified screening program was higher compared to the diseased group (*p* < 0.001). 40.9% (*n* = 95) of the non‐diseased carriers performed intensified breast diagnostics at the TUM. 16.4% were not included due to PBM, and the remaining 42.7% have either participated in the program at another hospital or not at all.

## 3 Discussion

In our study, we primarily sought to carry out a genotype–phenotype correlation in *BRCA1/2* carriers and in particular to demonstrate the effectiveness of prophylactic surgery.

With regard to the gPV *BRCA1* and *BRCA2* distribution, our patient collective showed a very similar distribution compared to existing data sets. 61.1% were *gBRCA1*, 38.6% were *gBRCA2* carriers (0.1% carrying both mutations; *n* = 1). Rebbeck et al. demonstrated almost identical prevalence rates when investigating more than 43,000 patients from the CIMBA database (Consortium of Investigators of Modifiers of *BRCA1/2*) [22]. When examining the higher percentage of gPV *BRCA1* carriers, it is important to address a potential bias. Specifically, in Germany, TNBC serves as a testing criterion for hereditary breast and ovarian cancer. As TNBC is frequently associated with gPV *BRCA1*, this could lead to an overestimation of the TNBC and gPV *BRCA1* rates respectively. In our study 55.3% of all breast cancer cases showed a triple negative tumor biology (85.1% associated to *BRCA1*), which does not reflect the normal subtype distribution.

Mean age at breast and ovarian cancer diagnosis overall correlates with other studies. In our research, breast cancer in gPV *BRCA1* carriers was diagnosed at a mean age of 41.6 and 46.1 years for gPV *BRCA2* carriers respectively. Very similar ages were found in other comprehensive studies [3, 23, 24]. Mean age for ovarian cancer diagnosis (*n* = 143) was 53.5 years for *gBRCA1* and 60.4 years for *gBRCA2* carriers. These ages were also very similar to a prospective cohort study [25].

We found that patients with a gPV in *BRCA1* had a higher percentage for mastectomies than patients with a gPV in *BRCA2* (45.4% vs. 38.9%; *p* = 0.171). This information should be interpreted with caution. On the one hand, the result was not statistically significant, and on the other, *BRCA1* carriers are generally more prone to develop more aggressive tumor types, such as TNBC, as well as having a higher histological grade at diagnosis, which may also influence surgical decision‐making [24, 26, 27].

Above all, in this study we were able to demonstrate the efficacy of prophylactic surgery in carriers with a gPV in the *BRCA1* and *BRCA2* gene. Over a median follow‐up period of 7 years up to a maximum of 44 years (95% CI: 6.70 to 7.70), we analyzed the prevalence of cancer after previous therapeutic and prophylactic surgery in a cohort of 875 female *BRCA1*/2 carriers. Despite the extremely high cancer risks that derive from a gPV in the *BRCA1* and *BRCA2* gene, none of the carriers who had previously undergone bilateral prophylactic mastectomy was diagnosed with breast cancer during the entire follow‐up period, emphasizing the effectiveness of prophylactic surgery. In addition to that the prevalence of breast cancer after contralateral prophylactic mastectomy was also very low, with a total of two cases. These numbers largely correlate with existing studies. In a prospective analysis by Domcheck et al., no breast cancer was detected after conducting PBM in *BRCA1/2* carriers during a 3‐year follow‐up (FUP) [14]. This data has been confirmed by another prospective study. Meijers‐Heijboer et colleagues stated to not have detected any breast cancer disease in the course of PBM after a mean FUP of 2.9 ± 1.4 years [16]. Critically considering the short time of FUP, Hartmann et al. were able to confirm these findings within 13 years of FUP [21]. In another study a 10‐year disease‐free survival of 100% was observed in the PBM group [19]. Rebbeck and colleagues supported these findings by demonstrating an incidence of 1.9% of breast cancer after a median follow‐up period of 6.4 years [18].

Within our analysis 17.9% of *gBRCA1* carriers and 15.2% of *gBRCA2* carriers developed contralateral breast cancer (CBC). Rhiem et al. calculated a cumulative 25‐year risk of CBC of 44.1% associated with a gPV in *BRCA1* and 33.5% with a gPV in *BRCA2* after initial diagnosis [28]. A prospective study showed a cumulative CBC risk of 25.1% and 6.6% for *gBRCA1* and *gBRCA2* carriers respectively (10 years after the first breast cancer) [29]. Yet, it was noticed that the risk of contralateral secondary disease after breast‐conserving surgery was considerably lower compared to mastectomy. The lower rate following a breast‐conserving approach could be attributed to scattered radiation after breast‐conserving therapy, though no statistically significant difference (*p* = 0.239) was found. While the concept of prophylactic mammary irradiation is currently being investigated to reduce the long‐term risk of contralateral breast cancer, this concept uses higher doses of radiation than the scattered radiation from primary cancer treatment [30, 31].

A systematic review and meta‐analysis of 13 studies demonstrated that *BRCA1/2* carriers had a significantly higher risk of ipsilateral breast cancer recurrence after breast‐conserving surgery than controls with sporadic breast cancer (RR: 1.59; *p* < 0.001) [32]. In our study we had data on 39 patients who developed ipsilateral breast cancer after breast‐conserving surgery (Table 1). The percentage of a second ipsilateral cancer (recurrence or secondary cancer) was lower when patients had first received a mastectomy than when patients had had a breast‐conserving surgery (27.7% vs. 37.5%; *p* = 0.255). Furthermore, our data suggest that mastectomy significantly lowers the rate of ipsilateral secondary carcinomas compared to ipsilateral recurrences (4.3% vs. 23.4%; *p* < 0.05). The oncological safety of breast‐conserving therapy in gPV *BRCA1*/2 carriers is currently still a controversial topic. Data on whether the risk for local recurrence after breast‐conserving therapy is elevated in comparison to mastectomy is contradictory. Shubeck et al. compared the locoregional occurrence rate among g*BRCA1/2* carriers who decided for breast‐conserving therapy versus mastectomy [33]. Yet there was no statistical significance. A meta‐analysis of four studies with 1254 patients found that g*BRCA1/2* carriers with breast‐conserving therapy had a significantly higher risk for local recurrence than patients with mastectomy [34].

Additionally, numerous studies have shown a reduced prevalence of ovarian cancer including secondary peritoneal carcinoma after PBSO [10, 11, 14]. However, there is still no effective measure to prevent primary peritoneal carcinoma, although the prevalence is very low. In Kauff et al., 1% of patients developed a peritoneal carcinoma after PBSO [11]. Another study registered 5 patients with intra‐abdominal carcinomatosis in 238 patients who had undergone PBSO (2.1%) [35]. The findings in our analysis were consistent with the existing data situation demonstrating a prevalence rate of 0.45% (*n* = 1) for carriers developing peritoneal carcinoma after PBSO.

Within the entire study collective, a total of 293 have undergone PBSO, with 6 women having an incidental finding of ovarian cancer and STIC respectively (=2.0%; 1.7% *gBRCA1* and 0.3% *gBRCA2*). In their analysis, Rebbeck et al. detected 8 women (3.1%) with ovarian or peritoneal carcinoma at the time of PBSO [10]. Finch et al. detected a much higher percentage of occult carcinoma findings at PBSO (6% of *BRCA1* carriers and 2% of *BRCA2* carriers) [36]. Currently PBSO is recommended from the age of 40, or 5 years before the youngest onset of ovarian cancer in the family [12, 15]. Emphasizing the patients with incidental findings of ovarian cancer in our study, all of them were older than 40 years when conducting PBSO (48–60 years), which raises the question of whether the findings could have been avoided by conducting prophylactic surgery earlier.

In addition to the prophylactic approaches, intensified breast cancer screening is of great importance in genetically predisposed women. In our study, only a small proportion, particularly of the breast cancer diseased *BRCA1*/2 carriers, have taken advantage of the intensified screening program. Nevertheless, our follow‐up data was limited. In this respect, it cannot be ruled out that the *BRCA1*/2 carriers of our study participated in the program at another center.

### 3.1 Jolie Effect

On 14.05.2013 Angelina Jolie published an article in the *New York Times*, where she addressed the identification of her gPV in the *BRCA1* gene and the prophylactic bilateral mastectomy she had undergone since [37]. The “Jolie effect” describes the effects the article has had since that in healthcare. A few studies stated an increase in contralateral risk‐reducing mastectomies and PBM after Jolies article [38, 39]. Two other studies could not detect an increase in mastectomy rates or the rate of PBM among women who had already been tested for a gPV in *BRCA1/BRCA2* [40, 41]. In correlation with the Jolie effect, we found a peak in the number of prophylactic mastectomies in 2013. After this first noticeable peak, mastectomy rates have risen since.

### 3.2 Limitations

As the study was executed with retrospective data over a long period of time, many patient cases were incomplete, and we did not have access to the same amount of data for each patient. We tried to obtain more current data on the patients through a follow‐up questionnaire, but even then, there were still many cases with incomplete data.

The effect of other treatments like chemotherapy or radiation was not included in the study. Especially when analyzing cancer recurrence, it should be taken into consideration that the surgical removal of the tumor is only one pillar of a systemic cancer therapy treatment.

Unfortunately, our data did not allow an analysis on the effect of PBSO on breast cancer. Further studies will have to be conducted to examine this point.

## 4 Materials and Methods

We included 875 women that were tested positive for gPV in *BRCA1* and *BRCA2* between 2002 and 2022 at the Center of Hereditary Breast and Ovarian Cancer (FBREK‐Center) of the Technical University Munich (TU Munich), Germany. The study is approved by the local Ethical Committee of the Technical University Munich (ID‐Nr. 2022‐476‐S‐KK) and written informed consent was obtained from all participants before entry into the study.

We collected retrospective data on genetic testing information and clinical data, as follows: age at diagnosis, tumor stage, tumor biology (e.g., estrogen/progesterone receptor status, Her2 status), type of surgery, previous cancer diseases and prophylactic surgeries. We also recorded age at the time of conducting prophylactic surgery (bilateral/contralateral PM; PBSO). Furthermore, we gathered information regarding the attendance at the intensified breast cancer screening program to evaluate the patients' compliance.

A follow up questionnaire was sent to 756 patients. Criteria for exclusion were death, unknown address, no consent for follow‐up questionnaires and residency in a non‐German speaking country. Response rate was 42%.

The beginning of follow‐up was marked by either a positive test for a gPV in *BRCA1/2* or a related cancer disease. Patients were censored at last contact or after responding to the follow‐up questionnaire. Mean follow up was 7.2 years (range 0–44 years).

SPSS 29.0.1.0 was used for all data analysis. Independent samples *t*‐tests and chi‐square tests were applied where appropriate. All statistical tests were considered significant if they met the criteria of a two‐sided hypothesis test with a *p*‐value < 0.05.

## Author Contributions

**Sabine Grill:** conceptualization, methodology, resources, writing – review and editing, supervision, project administration. **Sara Mendes:** methodology, investigation, formal analysis, writing – original draft, data curation, visualization. **Marion Kiechle:** conceptualization, methodology, resources, project administration, supervision. **Eva Maria Fallenberg:** resources, supervision. **Maryam Yahiaoui‐Doktor:** formal analysis.

## Funding

The authors have nothing to report.

## Ethics Statement

The study is approved by the local Ethical Committee of the Technical University Munich (ID‐Nr. 2022‐476‐S‐KK) and written informed consent was obtained from all participants before entry into the study.

## Conflicts of Interest

Sara Mendes, Maryam Yahiaoui‐Doktor and Eva Maria Fallenberg declare no conflicts of interest. Marion Kiechle: Renumeration: Springer Press, Biermann Press, Celgene, Astra Zeneca, Myriad Genetics, TEVA, Eli Lilly, GSK, Seagen, AllergoSan, FOMF, Roche, BESINS, Bayer AG. Consultant/Advisory Role: Myriad Genetics, Bavarian KVB, DKMS Life, BLAEK, TEVA, Exeltis, Roche, BESINS, Bayer AG. Equity owner: AIM GmbH, In Manas GmbH, Therawis Diagnostic GmbH. Funding: Sphingotec, Deutsche Krebshilfe, DFG, Senator Roesner Foundation, Dr. Pommer‐Jung Foundation, Waltraut Bergmann Foundation, Bavarian State Ministry of Economy, BMBF, Innovation Fond GBA. Sabine Grill: contractual fees with Astrazeneca, Roche, Daiichi Sankyo.

## Supporting information


**Dataset: S1** Anonymized dataset of healthy gPV BRCA1/2 carriers. Includes clinical parameters, mutation status, and surgical history for all study participants.

**Dataset: S2** Anonymized dataset of diseased gPV BRCA1/2 carriers. Includes clinical parameters, mutation status, and surgical history for all study participants.

## Data Availability

The data that supports the findings of this study are available in the Supporting Information of this article.

## References

[1] Robert‐Koch‐Institut , “Krebs in Deutschland für 2019/2020, Brustdrüse,” 2023.

[2] J. Ettl and R. Würstlein , Manual Mammakarzinom, Empfehlungen zur Diagnostik, Therapie, Nachsorge und Begleitung (W. Zuckschwerdt, 2023), 401.

[3] K. B. Kuchenbaecker , J. L. Hopper , D. R. Barnes , et al., “Risks of Breast, Ovarian, and Contralateral Breast Cancer for BRCA1 and BRCA2 Mutation Carriers,” JAMA 317, no. 23 (2017): 2402–2416. 28632866 10.1001/jama.2017.7112

[4] C. C. Riedl , N. Luft , C. Bernhart , et al., “Triple‐Modality Screening Trial for Familial Breast Cancer Underlines the Importance of Magnetic Resonance Imaging and Questions the Role of Mammography and Ultrasound Regardless of Patient Mutation Status, Age, and Breast Density,” Journal of Clinical Oncology 33, no. 10 (2015): 1128–1135. 25713430 10.1200/JCO.2014.56.8626 PMC5526626

[5] U. Bick , C. Engel , B. Krug , et al., “High‐Risk Breast Cancer Surveillance With MRI: 10‐Year Experience From the German Consortium for Hereditary Breast and Ovarian Cancer,” Breast Cancer Research and Treatment 175, no. 1 (2019): 217–228. 30725383 10.1007/s10549-019-05152-9

[6] F. Sardanelli , F. Podo , F. Santoro , et al., “Multicenter Surveillance of Women at High Genetic Breast Cancer Risk Using Mammography, Ultrasonography, and Contrast‐Enhanced Magnetic Resonance Imaging (The High Breast Cancer Risk Italian 1 Study): Final Results,” Investigative Radiology 46, no. 2 (2011): 94–105. 21139507 10.1097/RLI.0b013e3181f3fcdf

[7] I. J. Jacobs , U. Menon , A. Ryan , et al., “Ovarian Cancer Screening and Mortality in the UK Collaborative Trial of Ovarian Cancer Screening (UKCTOCS): A Randomised Controlled Trial,” Lancet 387, no. 10022 (2016): 945–956. 26707054 10.1016/S0140-6736(15)01224-6 PMC4779792

[8] S3‐Leitlinie Diagnostik, Therapie und Nachsorge maligner Ovarialtumoren2022. 161 p.

[9] S. S. Buys , E. Partridge , A. Black , et al., “Effect of Screening on Ovarian Cancer Mortality: The Prostate, Lung, Colorectal and Ovarian (PLCO) Cancer Screening Randomized Controlled Trial,” JAMA 305, no. 22 (2011): 2295–2303. 21642681 10.1001/jama.2011.766

[10] T. R. Rebbeck , H. T. Lynch , S. L. Neuhausen , et al., “Prophylactic Oophorectomy in Carriers of BRCA1 or BRCA2 Mutations,” New England Journal of Medicine 346, no. 21 (2002): 1616–1622. 12023993 10.1056/NEJMoa012158

[11] N. D. Kauff , J. M. Satagopan , M. E. Robson , et al., “Risk‐Reducing Salpingo‐Oophorectomy in Women With a BRCA1 or BRCA2 Mutation,” New England Journal of Medicine 346, no. 21 (2002): 1609–1615. 12023992 10.1056/NEJMoa020119

[12] R. Schmutzler and K. Rhiem , “Konsensusempfehlungen BRCA2,” (2022).

[13] A. Finch , M. Beiner , J. Lubinski , et al., “Salpingo‐Oophorectomy and the Risk of Ovarian, Fallopian Tube, and Peritoneal Cancers in Women With a BRCA1 or BRCA2 Mutation,” JAMA 296, no. 2 (2006): 185–192. 16835424 10.1001/jama.296.2.185

[14] S. M. Domchek , T. M. Friebel , C. F. Singer , et al., “Association of Risk‐Reducing Surgery in BRCA1 or BRCA2 Mutation Carriers With Cancer Risk and Mortality,” Journal of the American Medical Association 304, no. 9 (2010): 967–975. 20810374 10.1001/jama.2010.1237 PMC2948529

[15] K. Rhiem and R. Schmutzler , “Konsensusempfehlungen BRCA1,” 2022.

[16] H. Meijers‐Heijboer , B. van Geel , W. L. J. van Putten , et al., “Breast Cancer After Prophylactic Bilateral Mastectomy in Women With a BRCA1 or BRCA2 Mutation,” New England Journal of Medicine 345, no. 3 (2001): 159–164. 11463009 10.1056/NEJM200107193450301

[17] L. C. Hartmann , D. J. Schaid , J. E. Woods , et al., “Efficacy of Bilateral Prophylactic Mastectomy in Women With a Family History of Breast Cancer,” New England Journal of Medicine 340, no. 2 (1999): 77–84. 9887158 10.1056/NEJM199901143400201

[18] T. R. Rebbeck , T. Friebel , H. T. Lynch , et al., “Bilateral Prophylactic Mastectomy Reduces Breast Cancer Risk in BRCA1 and BRCA2 Mutation Carriers: The PROSE Study Group,” Journal of Clinical Oncology 22, no. 6 (2004): 1055–1062. 14981104 10.1200/JCO.2004.04.188

[19] B. A. M. Heemskerk‐Gerritsen , M. B. E. Menke‐Pluijmers , A. Jager , et al., “Substantial Breast Cancer Risk Reduction and Potential Survival Benefit After Bilateral Mastectomy When Compared With Surveillance in Healthy BRCA1 and BRCA2 Mutation Carriers: A Prospective Analysis,” Annals of Oncology 24, no. 8 (2013): 2029–2035. 23576707 10.1093/annonc/mdt134

[20] R. K. Alaofi , M. O. Nassif , and M. R. Al‐Hajeili , “Prophylactic Mastectomy for the Prevention of Breast Cancer: Review of the Literature,” Avicenna Journal of Medicine 8, no. 3 (2018): 67–77. 30090744 10.4103/ajm.AJM_21_18 PMC6057165

[21] L. C. Hartmann , T. A. Sellers , D. J. Schaid , et al., “Efficacy of Bilateral Prophylactic Mastectomy in BRCA1 and BRCA2 Gene Mutation Carriers,” JNCI Journal of the National Cancer Institute 93, no. 21 (2001): 1633–1637. 11698567 10.1093/jnci/93.21.1633

[22] T. R. Rebbeck , T. M. Friebel , E. Friedman , et al., “Mutational Spectrum in a Worldwide Study of 29,700 Families With BRCA1 or BRCA2 Mutations,” Human Mutation 39, no. 5 (2018): 593–620. 29446198 10.1002/humu.23406 PMC5903938

[23] N. Mavaddat , S. Peock , D. Frost , et al., “Cancer Risks for BRCA1 and BRCA2 Mutation Carriers: Results From Prospective Analysis of EMBRACE,” Journal of the National Cancer Institute 105, no. 11 (2013): 812–822. 23628597 10.1093/jnci/djt095

[24] N. Mavaddat , D. Barrowdale , I. L. Andrulis , et al., “Pathology of Breast and Ovarian Cancers Among BRCA1 and BRCA2 Mutation Carriers: Results From the Consortium of Investigators of Modifiers of BRCA1/2 (CIMBA),” Cancer Epidemiology, Biomarkers & Prevention 21, no. 1 (2012): 134–147. 10.1158/1055-9965.EPI-11-0775 PMC3272407 22144499

[25] J. Kotsopoulos , J. Gronwald , B. Karlan , et al., “Age‐Specific Ovarian Cancer Risks Among Women With a BRCA1 or BRCA2 Mutation,” Gynecologic Oncology 150, no. 1 (2018): 85–91. 29793803 10.1016/j.ygyno.2018.05.011

[26] L. Incorvaia , D. Fanale , M. Bono , et al., “BRCA1/2 Pathogenic Variants in Triple‐Negative Versus Luminal‐Like Breast Cancers: Genotype‐Phenotype Correlation in a Cohort of 531 Patients,” Therapeutic Advances in Medical Oncology 12 (2020): 1758835920975326. 33403015 10.1177/1758835920975326 PMC7747114

[27] M. J. Larsen , T. A. Kruse , Q. Tan , et al., “Classifications Within Molecular Subtypes Enables Identification of BRCA1/BRCA2 Mutation Carriers by RNA Tumor Profiling,” PLoS One 8, no. 5 (2013): e64268. 23704984 10.1371/journal.pone.0064268 PMC3660328

[28] K. Rhiem , C. Engel , M. Graeser , et al., “The Risk of Contralateral Breast Cancer in Patients From BRCA1/2 Negative High Risk Families as Compared to Patients From BRCA1 or BRCA2 Positive Families: A Retrospective Cohort Study,” Breast Cancer Research 14, no. 6 (2012): R156. 23216834 10.1186/bcr3369 PMC4053142

[29] C. Engel , C. Fischer , S. Zachariae , et al., “Breast Cancer Risk in BRCA1/2 Mutation Carriers and Noncarriers Under Prospective Intensified Surveillance,” International Journal of Cancer 146, no. 4 (2020): 999–1009. 31081934 10.1002/ijc.32396

[30] I. Shuryak , L. B. Smilenov , N. J. Kleiman , and D. J. Brenner , “Potential Reduction of Contralateral Second Breast‐Cancer Risks by Prophylactic Mammary Irradiation: Validation in a Breast‐Cancer‐Prone Mouse Model,” PLoS One 8, no. 12 (2013): e85795. 24376895 10.1371/journal.pone.0085795 PMC3869887

[31] D. J. Brenner , I. Shuryak , S. Russo , and R. K. Sachs , “Reducing Second Breast Cancers: A Potential Role for Prophylactic Mammary Irradiation,” Journal of Clinical Oncology 25, no. 31 (2007): 4868–4872. 17971581 10.1200/JCO.2007.11.0379

[32] M. Nara , S. Ishihara , A. Kitano , et al., “Does Breast‐Conserving Surgery With Radiotherapy in BRCA‐Mutation Carriers Significantly Increase Ipsilateral Breast Tumor Recurrence? A Systematic Review and Meta‐Analysis,” Breast Cancer 29, no. 3 (2022): 394–401. 35212965 10.1007/s12282-022-01343-3

[33] S. Shubeck , V. Sevilimedu , E. Berger , M. Robson , A. S. Heerdt , and M. L. Pilewskie , “Comparison of Outcomes Between BRCA Pathogenic Variant Carriers Undergoing Breast‐Conserving Surgery Versus Mastectomy,” Annals of Surgical Oncology 29, no. 8 (2022): 4706–4713. 35585432 10.1245/s10434-022-11756-1 PMC10161354

[34] C. Wang , Y. Lin , H. Zhu , et al., “Breast‐Conserving Therapy for Breast Cancer With BRCA Mutations: A Meta‐Analysis,” Breast Cancer 29, no. 2 (2022): 314–323. 34766244 10.1007/s12282-021-01312-2

[35] M. J. Casey , C. Synder , C. Bewtra , S. A. Narod , P. Watson , and H. T. Lynch , “Intra‐Abdominal Carcinomatosis After Prophylactic Oophorectomy in Women of Hereditary Breast Ovarian Cancer Syndrome Kindreds Associated With BRCA1 and BRCA2 Mutations,” Gynecologic Oncology 97, no. 2 (2005): 457–467. 15863145 10.1016/j.ygyno.2005.01.039

[36] A. Finch , P. Shaw , B. Rosen , J. Murphy , S. A. Narod , and T. J. Colgan , “Clinical and Pathologic Findings of Prophylactic Salpingo‐Oophorectomies in 159 BRCA1 and BRCA2 Carriers,” Gynecologic Oncology 100, no. 1 (2006): 58–64. 16137750 10.1016/j.ygyno.2005.06.065

[37] A. Jolie , My Medical Choice (2013), https://www.nytimes.com/2013/05/14/opinion/my‐medical‐choice.html.

[38] N. N. Basu , J. Hodson , S. Chatterjee , et al., “The Angelina Jolie Effect: Contralateral Risk‐Reducing Mastectomy Trends in Patients at Increased Risk of Breast Cancer,” Scientific Reports 11, no. 1 (2021): 2847. 33531640 10.1038/s41598-021-82654-x PMC7854742

[39] D. G. Evans , J. Wisely , T. Clancy , et al., “Longer Term Effects of the Angelina Jolie Effect: Increased Risk‐Reducing Mastectomy Rates in BRCA Carriers and Other High‐Risk Women,” Breast Cancer Research 17, no. 1 (2015): 143. 26603733 10.1186/s13058-015-0650-8 PMC4659163

[40] S. Desai and A. B. Jena , “Do Celebrity Endorsements Matter? Observational Study of BRCA Gene Testing and Mastectomy Rates After Angelina Jolie's New York Times Editorial,” BMJ 355 (2016): i6357. 27974323 10.1136/bmj.i6357 PMC5156611

[41] A. Liede , M. Cai , T. F. Crouter , D. Niepel , F. Callaghan , and D. G. Evans , “Risk‐Reducing Mastectomy Rates in the US: A Closer Examination of the Angelina Jolie Effect,” Breast Cancer Research and Treatment 171, no. 2 (2018): 435–442. 29808287 10.1007/s10549-018-4824-9 PMC6096880

